# Tracking effects of age of sign language acquisition and phonology in American Sign Language sentence processing

**DOI:** 10.3758/s13421-025-01695-z

**Published:** 2025-03-27

**Authors:** Anne Wienholz, Amy M. Lieberman

**Affiliations:** 1 Institute of German Sign Language and Communication of the Deaf, University of Hamburg, Gorch-Fock-Wall 7, 20354 Hamburg, Germany; 2 Wheelock College of Education and Human Development, Boston University, 2 Silber Way, Boston, MA 02215 USA

**Keywords:** American Sign Language, Eye tracking, Age of acquisition, Phonological processing, Phonological parameters

## Abstract

Processing sign language involves activation of phonological features of signs. Previous research provides evidence for effects of age of sign language acquisition as well as amount and type of phonological relatedness during processing of single signs, but it is unknown how these factors affect sentence-level sign processing. This paper presents a phonological priming eye tracking study of American Sign Language (ASL) processing, in which we systematically vary the degree and type of phonological relatedness in prime-target sign pairs embedded in ASL sentences. We tested degree of relatedness by using sign pairs sharing either one or two out of three phonological parameters. We tested type of relatedness by using signs that were phonologically related in all possible combinations of the parameters handshape, location, and movement. Participants were exposed to sign language either early (before the age of five years) or late (after the age of five years), allowing us to explore how age of sign language acquisition impacts activation of phonological features of signs. Late signers were more affected by the degree of relatedness than early signers; primes that shared any information with the target led to increased time to identify the target, regardless of the specific parameter(s) that overlapped. There was a high degree of variability for type of relatedness, but sign pairs that shared location were particularly salient. Group differences suggested varying sensitivities to phonological information in early and late signers. Our study emphasizes that phonological relatedness should be carefully controlled when examining sign processing in signers differing in their language backgrounds.

## Introduction

Most deaf individuals are born into hearing families and are not exposed to a fully accessible language from birth (Mitchell & Karchmer, 2004). These signers often experience delayed acquisition of a sign language. Previous research has shown that age of sign language acquisition has pervasive and long-lasting effects on various aspects of sign language comprehension and processing including syntax, morphology and phonology (Boudreault & Mayberry, 2006; Cheng & Mayberry, 2019; Hall et al., 2012; Mayberry et al., 2002; Mayberry & Eichen, 1991; Mayberry & Fischer, 1989). Age of sign language acquisition effects have also been found to have persistent effects on the organization of the mental lexicon, especially with respect to how phonological information is accessed during sign comprehension. In the current study, we investigate phonological processing using eye tracking in signers differing in their age of acquisition of American Sign Language (ASL) and how that affects their sensitivity to sub-lexical features of the language. Understanding the way in which phonological processing differs in signers based on their early input can shed light on the role of early language experience in shaping the organization of the mental lexicon.

Processing language in any modality requires individuals to access their mental lexicon to retrieve information about the perceived input. When perceiving words, individuals will show activation for both the words being perceived, as well as words that overlap in phonological, semantic, and other information with the target word. Phonological processing has been investigated by presenting individuals with pairs of phonologically related words in a range of experimental paradigms, including priming. In spoken language priming studies, words are typically recognized faster when preceded by a phonologically related word than when preceded by an unrelated word. Moreover, differences have been observed in the speed and accuracy of word recognition based on the degree of relatedness between the prime and target words, and the position of overlap within the word such as differences for onset and rhyme (Hillinger, 1980; Radeau et al., 1995; Slowiaczek et al., 2000; Slowiaczek & Hamburger, 1992; Slowiaczek & Pisoni, 1986).

### Phonological processing in sign language

In sign perception, signs that share phonological information are those that are related in one or more phonological parameters. The three main parameters are typically described as handshape, location and movement (Stokoe, 1960; Stokoe et al., 1965). However, researchers vary in how they define phonological relatedness. While some studies define phonological competitors as signs that *share* a specific parameter, others define them as sign pairs that share two parameters and *differ* along one parameter. The varied methodological approaches to comparing signs based on degree of phonological relatedness make it challenging to draw widespread conclusions about its effects on sign processing. A summary of previous findings in early signers related to facilitation and inhibition by parameter is presented in Table 1 (adapted from Wienholz et al., 2021).

**Table 1:** Effects of phonological priming in early signers (adapted from Wienholz et al., 2021)

Task	Language	Shared parameter					
		HS	LOC	MOV	HS+MOV	HS+LOC	LOC+MOV
Lexical decision	ASL (Corina & Hildebrandt, 2002)	—	inhibition	inhibition	—	—	—
	ASL (Corina & Emmorey, 1993)	no effect	inhibition	facilitation	—	—	—
	ASL (Mayberry & Witcher, 2005)	—	—	—	no effect (no parameter-based analysis)		
	LSE (Carreiras et al., 2008)	no effect	inhibition	—	—	—	—
	BSL (Dye & Shih, 2006)	no effect	facilitation	no effect	no effect	no effect	facilitation
ERP	LSE (Gutiérrez et al., 2012)	inhibition	inhibition	—	—	—	—
	DGS (Hosemann et al., 2020)	—	—	—	facilitation (no parameter-based analysis)		
	ASL (Meade et al., 2018)	—	—	—	facilitation (no parameter-based analysis)		
	ASL (Meade et al., 2021, repetition task)	facilitation	facilitation	—	—	facilitation	—
	ASL (Meade et al., 2021, categorization task)	no effect	inhibition	—	—	facilitation	—
Eye tracking	BSL (Thompson et al., 2013)	—	—	—	late facilitation	no effect	facilitation
	ASL (Lieberman et al., 2015)	—	—	—	facilitation	no effect	inhibition
	DGS (Wienholz et al., 2021)	—	—	—	facilitation	no effect	no effect

Abbreviations: Parameter: HS = handshape; LOC = location, MOV = movement; HS+MOV = handshape and movement; HS+LOC = handshape and location; LOC+MOV = location and movement; Languages: ASL = American Sign Language; BSL = British Sign Language; DGS = German Sign Language; LSE = Spanish Sign Language

Sign language processing has been investigated using a range of paradigms. Results from primed lexical decision tasks in sign languages suggest that phonological relatedness inhibits, rather than facilitates, sign recognition. Signers were slower to recognize signs when presented with primes that shared location or movement with the target sign (Carreiras et al., 2008; Corina & Emmorey, 1993; Corina & Hildebrandt, 2002). Sign phonological processing has also been studied using eye tracking tasks of sign recognition (Lieberman et al., 2015; Thompson et al., 2013; Wienholz et al., 2021). Thompson et al. (2013) explored the nature of lexical access in British Sign Language (BSL) using the visual world paradigm. They presented signers with a BSL video (*I see ‘target sign’*) surrounded by four pictures and instructed them to indicate via key press whether the target sign was presented in one of the pictures. On the critical trials, the target was not present, and the distractors contained a semantically and a phonologically related picture that shared two out of three parameters with the target. Thompson et al. (2013) observed no differences in accuracy across conditions, but they did see both inhibitory and facilitative effects of phonological activation when looking at response times to the phonologically related competitors. The direction of effects varied according to where in the time course (early vs late) they were measured, and which phonological parameters were shared. Overall, facilitation was observed when movement was shared, suggesting that signers used this visually salient information during lexical access even though the movement parameter is identifiable later compared to the handshape and location parameters.

Beyond studies of single sign recognition, it is important to use stimulus material that reflects the natural use of everyday language to get a better understanding of natural language processing. Wienholz et al. (2021) investigated the role of phonological priming in signers perceiving prime-target pairs embedded in sentences. In an eye tracking paradigm, they presented early signers, who acquired German Sign Language (DGS) before the age of five years, with DGS sentences containing prime and target sign pairs sharing two out of three parameters. DGS signers showed a facilitation effect when signs shared handshape and movement as reflected in increased target fixations in these trials compared to their matched unrelated trials. However, the authors noted that it remained unclear whether observed effects were due to sharing two parameters or due to differing in the remaining one parameter. The current study follows a similar approach used in Wienholz et al. (2021) but includes sign pairs that share one and two out of three parameters.

A variety of tasks have also been presented to signers while neural responses were measured via EEG, showing facilitation for processing related signs sharing two out of three parameters compared to unrelated signs reflected in reduced N400 amplitudes (Hosemann et al., 2020; Meade et al., 2018). A recent study by Meade et al. (2021) aimed to identify the role of specific parameter relatedness in sign processing in an EEG setting by including signs sharing handshape only, location only, or both handshape and location. Participants were presented with sign pairs varying in their phonological relation and completed a repetition detection task as well as a semantic categorization task. Meade et al. (2021) observed an overall facilitation effect for shared handshape and location as well as shared handshape. However, the effect for shared handshape was task-dependent and was only present in the repetition detection task. The effects for shared location were also task-dependent in that the two tasks evoked opposite patterns. Again, these results suggest different roles of each parameter during sign recognition.

### Phonological processing and age of sign language acquisition

The age of acquiring a sign language has been shown to affect some, but not all, aspects of phonological processing. For example, in a gating task, early and late signers showed the same order of parameter identification with location first, followed by handshape shortly after and movement last. Signers only differed in the timing of identification for the movement parameter, which was identified faster by early signers than by late signers (Emmorey & Corina, 1990). Moreover, parameter identification was faster in early signers compared to late signers when context was provided (Clark & Grosjean, 1982). Factors such as lexical familiarity and phonological neighborhood were shown to affect sign language recognition speed and accuracy differently in early and late signers (Carreiras et al., 2008; Caselli et al., 2021; Morford & Carlson, 2011).

To date, only a few sign language studies using a priming paradigm have demonstrated priming effects (i.e., facilitation or inhibition) in which early and late signers have performed differently. Table 2 presents a summary of prior studies and their effects for late signers. Using a primed lexical decision task, Mayberry & Witcher (2005) examined the processing of sign pairs that shared two out of three phonological parameters in early and late signers of ASL. The data showed no priming effects for signers acquiring ASL from birth, but reaction times increased when age of ASL acquisition increased, suggesting that age of sign language acquisition influences the way the lexicon is accessed. Dye & Shih (2006) systematically investigated the different parameter and parameter combinations in a lexical decision task conducted with early and late signers of British Sign Language (BSL). The groups did not differ in accuracy, but early signers were overall faster than late signers to respond. Furthermore, while early signers replied faster to sign pairs that shared location or location and movement, late signers were faster for pairs that shared movement. Gutiérrez et al. (2012) examined processing of signs and non-signs sharing handshape or location in early and late signers of Spanish Sign Language (LSE) using electroencephalography (EEG). The form-based priming and lexical decision task showed inhibition effects for both parameters but with a different timing in the EEG data suggesting different roles for handshape and location during lexical access. However, effects were stronger in early than in late signers which the authors interpret as less efficient processing in late signers (Gutiérrez et al., 2012).

**Table 2:** Effects of phonological priming in late signers

Task	Language	Shared parameter					
		HS	LOC	MOV	HS+MOV	HS+LOC	LOC+MOV
Lexical decision	ASL (Mayberry & Witcher, 2005)	—	—	—	inhibition (no parameter-based analysis)		
	BSL (Dye & Shih, 2006)	no effect	no effect	facilitation	no effect	no effect	no effect
ERP	LSE (Gutiérrez et al., 2012)	inhibition	inhibition	—	—	—	—
Eye tracking	ASL (Lieberman et al., 2015)	—	—	—	no effect	no effect	no effect

Abbreviations: Parameter: HS = handshape; LOC = location, MOV = movement; HS+MOV = handshape and movement; HS+LOC = handshape and location; LOC+MOV = location and movement; Languages: ASL = American Sign Language; BSL = British Sign Language; LSE = Spanish Sign Language

The effect of age of sign language acquisition was systematically investigated using a visual world eye tracking paradigm in the study by Lieberman et al. (2015, 2016). They presented early signers, who were exposed to ASL before age two, and late signers, who learned ASL between ages five to 14 years, with videos of single ASL signs surrounded by four pictures, including a picture whose sign was a phonological competitor of the target sign. Both early and late signers looked more to the phonological distractors than they did to unrelated distractors, but only early signers also showed fewer looks to the target picture in the presence of phonological competitors. This pattern suggests that late signers did indeed recognize the phonological relationship, but this did not affect their overall looking pattern to the target sign.

In summary, previous research suggests that age of sign language acquisition is a factor affecting phonological processing in signers. However, the variation in how phonological similarity is defined, along with differences in task demands and group definitions, has led to complex and inconsistent findings regarding how phonology and age of sign language acquisition interact in sign processing.

### The current study

Two factors seem important in understanding phonological priming effects in sign languages: the degree of relatedness (i.e., how many parameters are shared) and the type of relatedness (i.e., which parameters are shared). The current study aimed to investigate these two factors by systematically varying the phonological relationship in sign pairs embedded in ASL sentences. We examine all possible parameter combinations and their effects during sentence-level processing. In addition, given the established relationship between age of sign language acquisition and phonological processing, we compare signers who were exposed to ASL early (i.e., before the age of five years), and signers who were exposed to ASL late (i.e., after the age of five years).

To investigate phonological priming, we recorded deaf signers’ eye movements while presenting them with ASL sentences containing sign pairs that varied in the degree (i.e., one or two shared parameters) and type (i.e., which parameter(s) are shared) of phonological relationship between prime and target sign. Participants were asked to select via key press the picture matching the ASL sentence. First, we predicted that early signers would have more efficient lexical recognition, and thus would be faster to recognize the target across conditions compared to late signers. Second, for both early and late signers, we expected to find the strongest priming effects for sign pairs that had a high degree of relatedness; thus, we expected to see larger effects for signs that share two parameters than for signs that share only one parameter. Specifically, we predicted that signers would be faster to recognize the target sign and would spend more time overall fixating the target when it was preceded by a phonologically related sign. We speculated that this effect would be greatest when the phonologically related sign had the highest degree of relatedness with the target (i.e., two shared parameters). Third, we predicted that there might be an interaction effect, such that early and late signers show differential effects for sign pairs with greater phonological relatedness, reflected in differences in the speed and accuracy of target fixations between groups. Finally, we sought to explore whether specific parameter combinations would drive priming effects in early and late signers, but we did not have specific predictions about which parameter combinations would impact sign recognition, so our investigation of type of relatedness is exploratory.

## Methods

### Participants

Participants were 48 deaf and hard of hearing adult signers of ASL. Signers were grouped according to the age of first exposure to ASL as self-reported on a language background questionnaire: *early signers* (*N* = 24) were first exposed to ASL before 5 years of age; *late signers* (*N* = 24), were first exposed to ASL after the age of 5 years (see Table 3 below for demographic information). We chose five years as the dividing age because this age is clearly past the initial sensitive period for language acquisition, and because age five is when many children start formal schooling. As is typical among the population of deaf and hard of hearing adults with variable ages of exposure to ASL, participants in the late signing group were heterogeneous in terms of their self-ranked ASL fluency. Participants were recruited via advertisements (online and on paper) in the Northeast US, specifically targeting programs serving deaf individuals.

**Table 3:** Overview of participants’ demographic information for early and late signers

	Early signers (N=24)	Late signers (N=24)
**Age (in years)**		
Mean (SD)	29.7 (11.2)	25.1 (6.49)
Median [Min, Max]	26.0 [20.0, 60.0]	22.0 [18.0, 38.0]
**Gender**		
Female	14 (58.3%)	15 (62.5%)
Male	10 (41.7%)	9 (37.5%)
**Age of ASL acquisition (in years)**		
Mean (SD)	0.25 (0.74)	16.4 (4.55)
Median [Min, Max]	0 [0, 3.00]	17.0 [7.00, 25.0]
**Years of exposure to ASL**		
Mean (SD)	29.4 (11.3)	8.71 (9.32)
Median [Min, Max]	26.0 [17.0, 60.0]	5.50 [< 1, 31.0]
**ASL fluency (self-rating;** **1 = “I am fluent in ASL”;** **7 = “I do not use ASL”)**		
Mean (SD)	1.14 (0.35)	3.08 (1.74)
Median [Min, Max]	1.0 [1.0, 2.0]	3.0 [1.0, 6.0]
Missing	2 (8.3 %)	0 (0 %)
**Hearing status**		
Deaf (severe/profound)	20 (83.3%)	10 (41.7%)
Hard of Hearing (mild/moderate)	4 (16.7%)	14 (58.3%)
**Race**		
Native American/Alaskan	1 (4.2%)	0 (0%)
White	22 (91.7%)	12 (50.0%)
African American/Black	0 (0%)	9 (37.5%)
Asian	0 (0%)	1 (4.2%)
More than one race	1 (4.2%)	1 (4.2%)
Other	0 (0%)	1 (4.2%)
**Ethnicity**		
Hispanic or Latina/o/x	1 (4.2%)	4 (16.7%)
Not Hispanic or Latina/o/x	22 (91.7%)	14 (58.3%)
Missing	1 (4.2%)	6 (25.0%)
**Highest level of education**		
Doctorate	1 (4.2%)	1 (4.2%)
Masters	3 (12.5%)	4 (16.7%)
College degree	10 (41.7%)	4 (16.7%)
Some college	8 (33.3%)	7 (29.2%)
High school degree	2 (8.3%)	7 (29.2%)
No high school degree	0 (0%)	1 (4.2%)

Table created using the Table 1 package in R (Rich, 2023)

Six additional adults were tested but had to be excluded because they were unable to finish the task due to technical issues (*N* = 3), they did not reach the trial count of at least three trials for each parameter or parameter combination (*N* = 2) or were missing demographic information (*N* = 1). All participants viewed study information in ASL and/or written English and gave written consent prior to the experiment. All communication between experimenter and participants took place in ASL. The study was approved by the Boston University Institutional Review Board.

### Experimental design

**Fig. 1:**
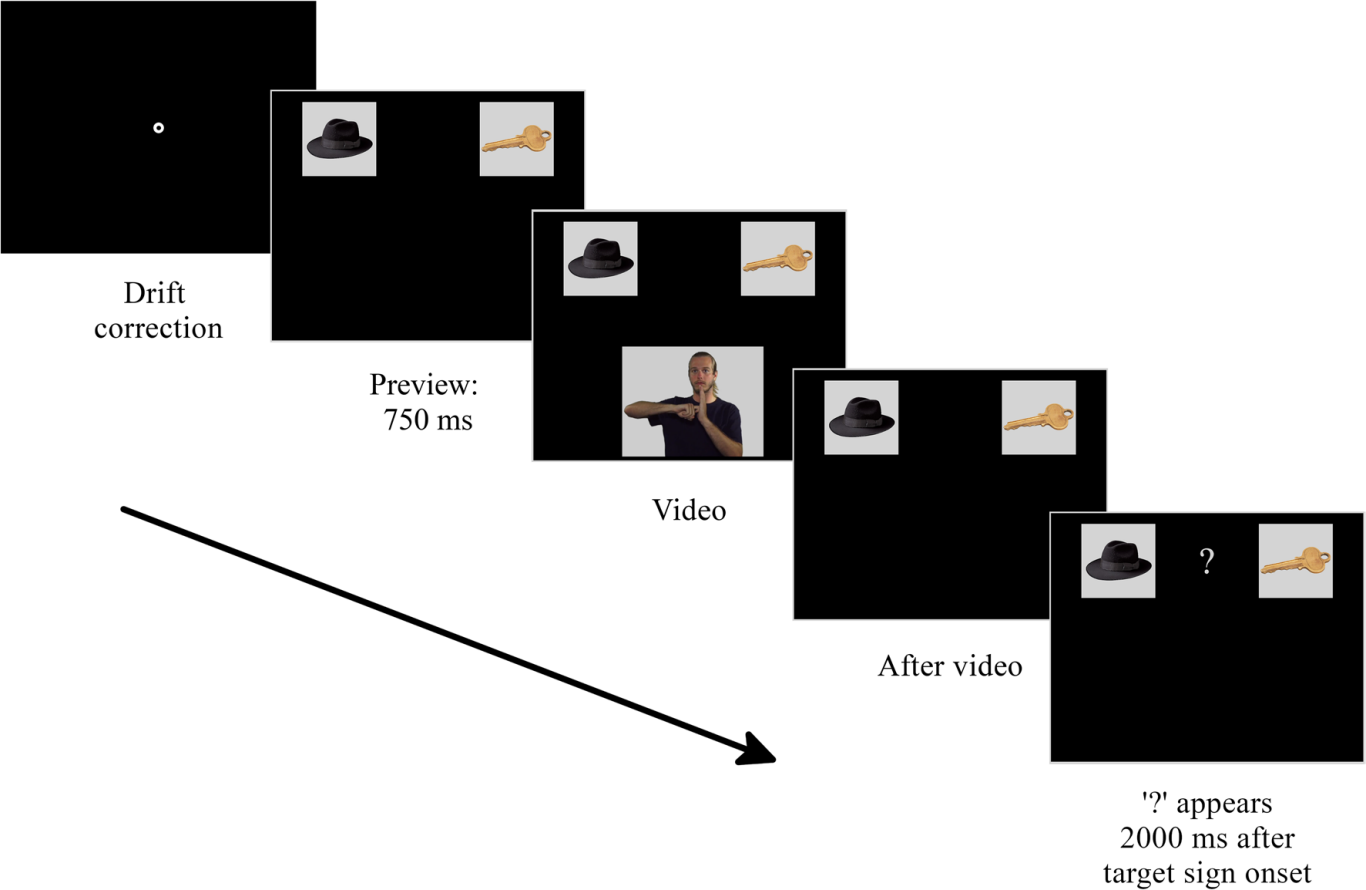
Structure of a single trial

The structure of the experiment was a two-alternative choice design: participants viewed pictures of concrete objects, followed by a video of an ASL sentence. Trials started with the presentation of two pictures at the top of the screen. Then the ASL video appeared below the pictures. The video disappeared at the end of the sentence and participants were prompted to select a picture with a question mark that appeared between the pictures 2000 ms after target sign onset. The extended time between the target and the question mark was included as part of the adaptation of the visual world paradigm to sign language. Specifically, it allows participants to sequentially perceive the pictures, the sentences and then the pictures again. This provides participants with enough time to shift their gaze to the pictures in response to the linguistic stimulus. On each trial, eye movement recordings started at the initial presentation of the pictures and continued until participants selected a picture (see Fig. 1).

### Experimental conditions

The final sign in each sentence corresponded to one of the pictures (i.e., the target). Each sentence also contained a phonologically related sign, which was separated from the target sign by a filler sign (e.g., and, index, or).^1^ Grammatically, the sentences all followed a consistent structure of SUBJECT VERB OBJECT1[prime] AND OBJECT2[target]. (e.g., my bag inside i have apple[prime] and key[target]). Some sentences also began with a time or location expression (e.g., yesterday, my bag inside) to provide a setting for the upcoming sentence.

**Fig. 2:**
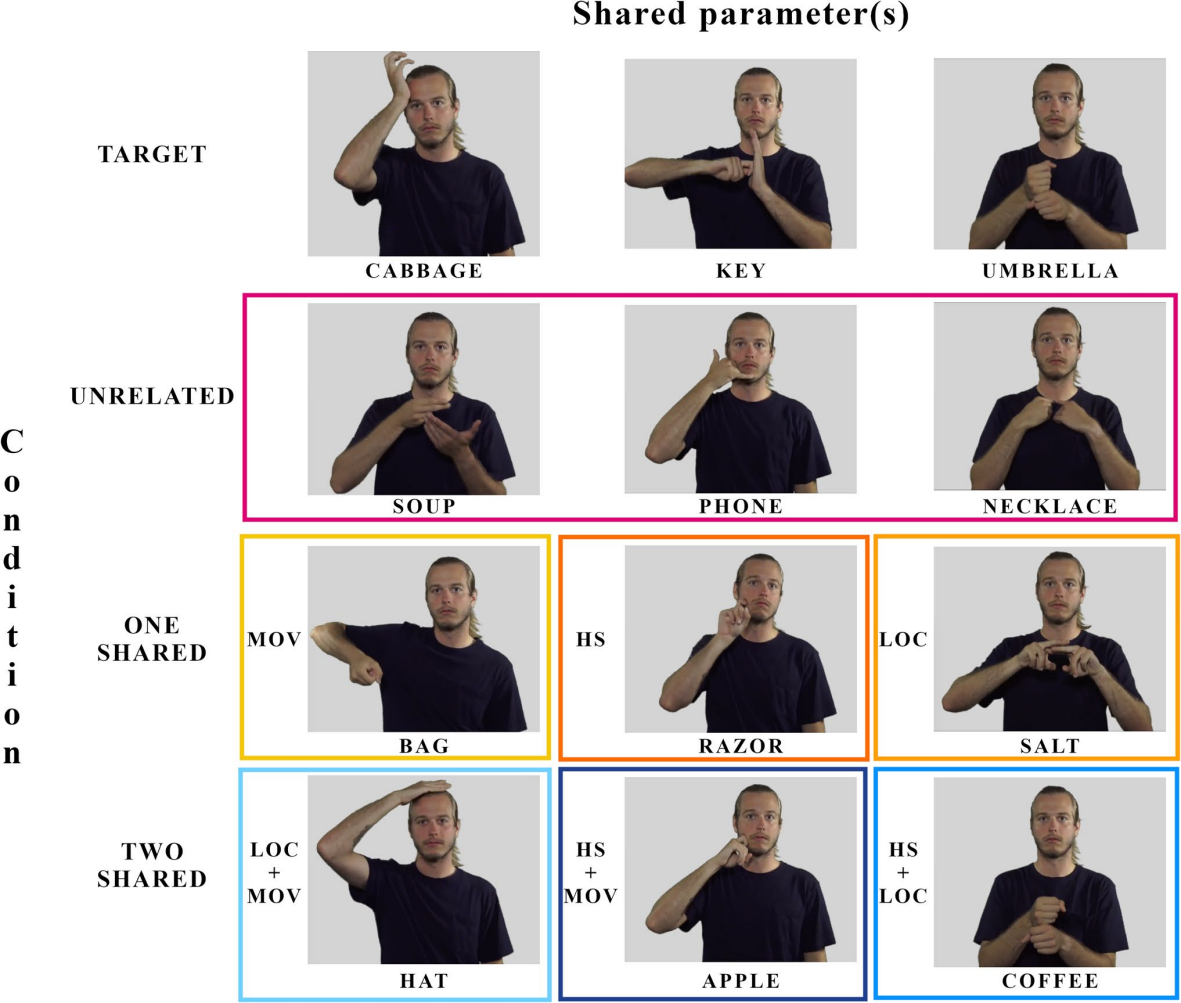
Example of sign pairs in each condition. The target is shown in the uppermost row. Conditions are presented as rows (from top to bottom): unrelated, one shared parameter and two shared parameters. Within the two bottom rows, the shared parameter or parameter combinations are presented and indicated left to the picture. Colored frames correspond to different conditions (pink: unrelated; orange: one shared parameter; blue: two shared parameters) and different shades of these colors denote the different parameters. This color coding is used in subsequent plots. For each sign pair, the target is combined with one of the signs within the same column

The prime and target signs systematically varied in the degree and type of related phonological parameters (handshape, location, and movement). We defined shared parameter according to the primary handshape (of the dominant hand), movement, and major location. In the *two shared parameters* condition, the phonologically related sign shared two parameters with the target and differed by only one parameter. Pairs included each possible combination of parameters: handshape and movement (HS+MOV), handshape and location (HS+LOC) or location and movement (LOC+MOV). In the *one shared parameter* condition, the phonologically related sign shared a single parameter with the target: handshape (HS), location (LOC) or movement (MOV). In the *unrelated* or control condition, there was no phonological relationship between the sign pairs (see Fig. 2). We used ASL-LEX (Sehyr et al., 2021) to determine the phonological parameters for each sign. Across conditions, sign pairs were unrelated semantically (i.e., prime and target did not belong to the same semantic category) and unrelated phonologically in written English. Prime signs were not controlled a priori for frequency, phonological neighborhood density or iconicity. However, we subsequently analyzed these factors for the prime signs using the ratings provided in ASL-LEX (Sehyr et al., 2021). We ran t-tests on prime signs between conditions; mean frequency and phonological neighborhood density did not differ between conditions (frequency: all p > .23; phonological neighborhood density: all p > .09). For iconicity, we only had iconicity ratings for a subset (70%) of the signs. Among that subset, signs in the two-shared parameter condition were more iconic than signs in the unrelated condition (p = .04). There were no other significant differences in iconicity of signs between conditions. See the Supplemental Material A on OSF (https://osf.io/esvr2/) for a complete list of the stimulus material and videos.

In total, there were 90 trials with 30 trials in each condition. Within each condition, there were ten trials per parameter or parameter combination. Each target sign appeared in each condition, but with a unique paired sign. For example, the target sign key appeared in the two shared parameter condition with apple (shared HS+MOV), in the one shared parameter condition with the sign RAZOR (shared HS), and in the unrelated condition with the sign phone (no shared parameters) (Table 4).

**Table 4:** Example stimulus for each condition. Critical sign pairs are marked in bold

Condition	Shared parameter	Example stimulus
Unrelated	None	shelf there i have index **phone** ix **key** ‘On the shelf, I have there a **phone** [and] there a **key**.’
One shared parameter	Handshape	my neighbor look-for **razor** and **key** ‘My neighbor looks for a **razor** and a **key**.’
Two shared parameters	Handshape+movement	my bag inside i have **apple** and **key** ‘In my bag, I have an **apple** and a **key**.’

Overall, we selected frequent signs (based on subjective judgment) and avoided signs that have high dialectical variation. An additional set of four sentences with the same structure as the stimulus sentences were used as practice items.

### Stimuli creation

The pictures were color photo-realistic 300 x 300 pixel presented on a light grey background at the top of the screen. The pictures displayed the target and an unrelated distractor.^2^ The videos were 550 x 450 pixels with a light grey background. The video was presented below the pictures to ensure that pointing signs in the sentences, which typically have a downward orientation, were not interpreted as directional pointing towards one of the pictures (Wienholz & Herrmann, 2023).

The ASL sentences were recorded by a deaf native signer with reduced non-manuals and reduced signing speed. Each video was edited to start one frame before the signer moved from a resting position to initiate the sign, and end one frame after the signer returned to a resting position next to the body. While the initial portion of the sentence varied slightly in timing, the final three signs (prime, filler sign, target) were edited to have consistent timing: the prime and filler were edited to be exactly 55 frames (1646.5 ms) long by adjusting the video speed slightly up or down, based on the average length of the stimuli.^3^ The first frame was the frame when the target handshape of the sign was completely recognizable, regardless of target orientation or location (Wienholz et al., 2018) and the final frame was the end of the intermediate sign before the signer’s hand transitioned to the target sign. The sentence-final target sign was edited to have a length of exactly 22 frames (724 ms), also based on the average length of the stimuli. The target handshape again had to be recognizable in the first frame and then sign offset was used as the last frame (i.e., last frame of the sign’s final hold). The videos were pilot tested with a small group of signers, and none perceived the adjustment of video speed.

### Procedure

After obtaining written signed consent, participants were seated at a table in front of a 17-inch computer screen with a resolution of 1280x1024 pixels. The Eyelink 1000 Plus eye-tracker (SR Research Ltd.) in a remote desktop configuration was placed below the screen approximately 580–620 mm from the participants’ face and recorded eye movements at 500 Hz. Participants saw a pre-recorded ASL instruction video, followed by the trials. At the end of each trial, participants pressed a key corresponding to the left picture, right picture or ‘I don’t know’ if they were unsure or missed the video. Prior to the start of the experiment, a 5-point calibration and validation sequence were performed. Additionally, a drift correction check was completed before each trial. Data were binned offline into 30 ms sequences.

Stimuli were presented in three blocks of 30 trials preceded by a block of four practice items to familiarize participants with the procedure. Each sign pair only occurred once per block. The order of blocks was counterbalanced. The order of trials within blocks was randomized with a maximum of two consecutive trials of the same condition. Additionally, the position of the pictures was counterbalanced within each list. Overall, the study took about 15–20 minutes to complete.

### Analysis approach

All analyses were done using R (Version 4.3.0, R Core Team, 2023) with the packages eyetrackingR (Forbes et al., 2021), boot (Canty & Ripley, 2024) and lme4 (Bates et al., 2015) as well as sjPlot (Lüdecke, 2023) to generate model outputs. All R scripts used in the analysis are provided on OSF (https://osf.io/esvr2/). We assessed accuracy according to which picture the participants selected. Eye movement analyses were only conducted on trials for which participants selected the target picture, which resulted in removing 109 (2.4%) trials (excluded trials for early signers: unrelated – 4, one shared – 4, two shared – 10; excluded trials for late signers: unrelated – 30, one shared – 32, two shared – 29). Next, we excluded trials that exceeded the trackloss threshold of 50% (i.e., we removed trials where participants were not fixating the areas of interest for at least 50% of the trial) leading to the removal of an additional 507 (11.5%) trials (excluded trials for early signers: unrelated – 103, one shared – 86, two shared – 92; excluded trials for late signers: unrelated – 80, one shared – 70, two shared –76). Finally, we excluded two participants, both early signers, who did not reach the trial count of at least three trials for each parameter or parameter combination as described above in the Participants section.

Analyses included the picture selection (via key press) and eye movements. Regarding picture selection, we analyzed accuracy and reaction time to select the target. Eye movement data were analyzed according to fixations to three areas of interest (AOI) (i.e., the video and the two pictures) excluding non-AOI looks. We included a time course analysis and a window analysis. For the time course, we ran a divergence point analysis to determine the earliest divergence between target and distractor fixations allowing us to estimate the onset of a priming effect (Ito & Knoeferle, 2023; Stone et al., 2021). We computed a log-ratio between target and distractor fixations to capture a fixation bias approach and to test when this measure significantly deviates from zero. We then applied a non-parametric bootstrapping approach that accounts for potentially non-normally distributed data. Note that in the bootstrapping approach, the divergence point slightly varies each time the bootstrapping function is executed. This is approach includes two steps: (1) determining the divergence point for each group or condition, and then (2) identifying the difference in divergence points between groups or conditions respectively (Stone et al., 2021). This distribution of differences, as indicated by the confidence interval, is then used to assess the reliability of effects. Thus, a confidence interval that does not contain zero indicates a reliable difference between the groups or conditions tested. The time course included fixations from target sign onset until 2000 ms following target sign offset.

All window analyses included overall fixations in a 1000 ms time window starting 200 ms after target sign offset to ensure that gaze shifts were in response to the target. To analyze gaze in the window analyses, we calculated gaze fixations as follows: log[(proportion target looks + 1) / (proportion distractor looks + 1)]. This transformation allowed to assess participants’ target looking relative to the distractor while reducing noise (i.e., looking offscreen or looking to the video). Additionally, natural log transformation was applied for statistical integrity as this supported approaching a normal distribution. Transformed positive values indicated more looks to the target while negative values indicated more looks to the distractor. In the following Results section, each analysis begins with a comparison of early vs. late signers, followed by separate analyses in each group to examine effects of degree and type of relatedness.

## Results

### Picture selection

We first assessed accuracy and speed in selecting the target picture (Table 5). Both groups were highly accurate in choosing the target. We used a mixed-effects model to test for effects of group and condition as well as their interaction with participants as random effects. Analyses showed no effect of group and condition and no interaction (all *p* > .2) regarding picture selection accuracy.

**Table 5:** Mean percent accuracy in picture selection by condition for early and late signers

Condition	Early signers	Late signers
Total	99.3 %	95.6 %
Unrelated	99.51 %	95.65 %
One shared parameter	99.51 %	95.36 %
Two shared parameters	98.77 %	95.80 %

**Fig. 3:**
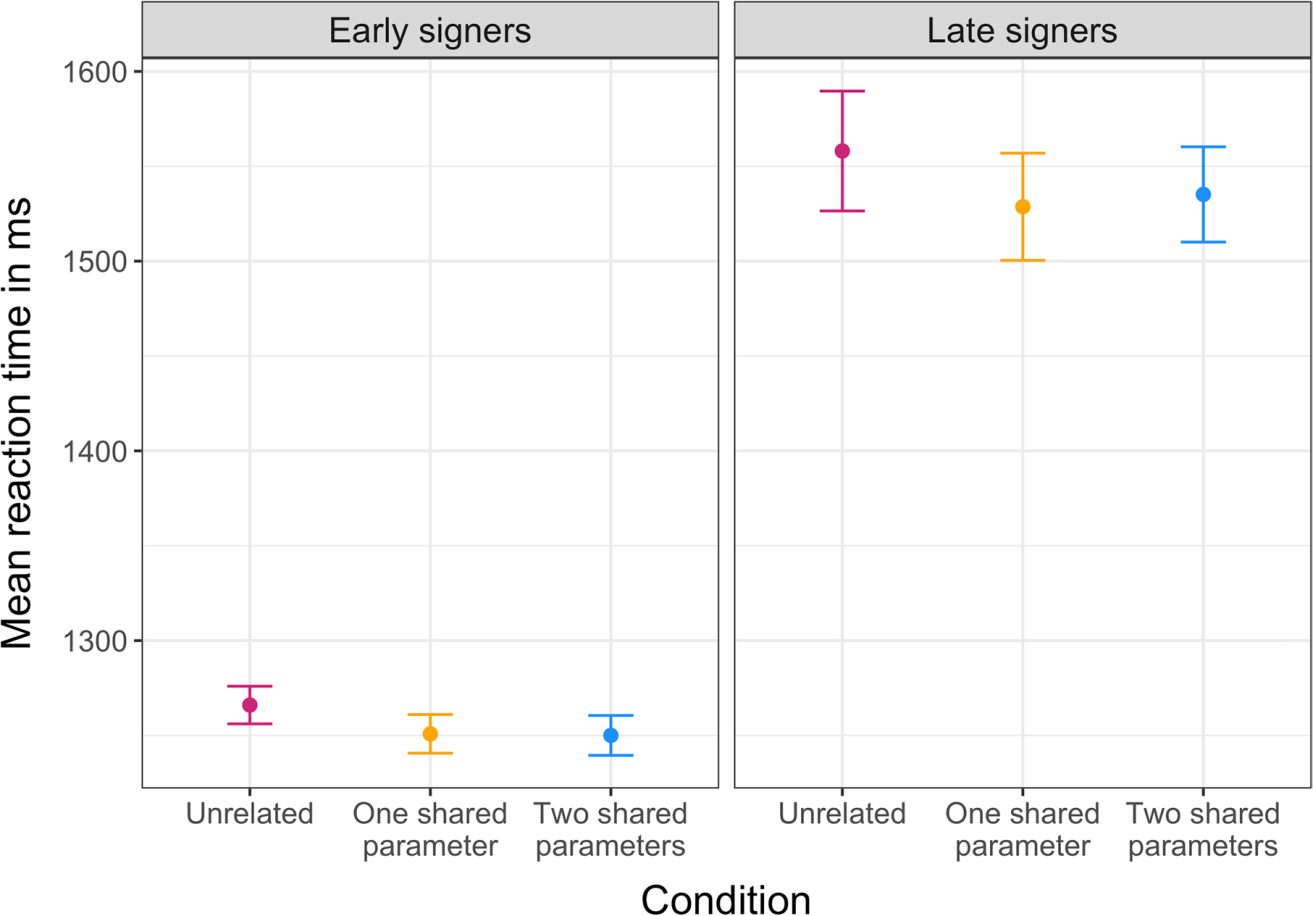
Mean reaction time of target selection (key press) for early signers (left column) and late signers (right column) and condition (on the x-axis in each panel from left to right: unrelated – pink, one shared parameter – orange, two shared parameters – blue). Errors bars indicate standard error

Next, we analyzed reaction time to select the target picture via a key press, starting from the appearance of the question mark, which initiated participants’ responses (Fig. 3). We predicted faster reaction times by early vs late signers, and faster reaction times for targets that were preceded by phonologically related signs. A mixed-effects model including group and condition as well as their interaction as fixed effect and participants as random effects revealed a main effect of group (*ß* = 297.50, 95% CI [178.52, 416.47], *p* < .001, *R*^*2*^ = .074), but no effect of condition and no interaction (*p* > .5). Thus, early signers were faster in target selection than late signers across conditions, but phonological relatedness did not predict reaction time.

### Eye tracking data

#### Degree of relatedness

**Fig. 4:**
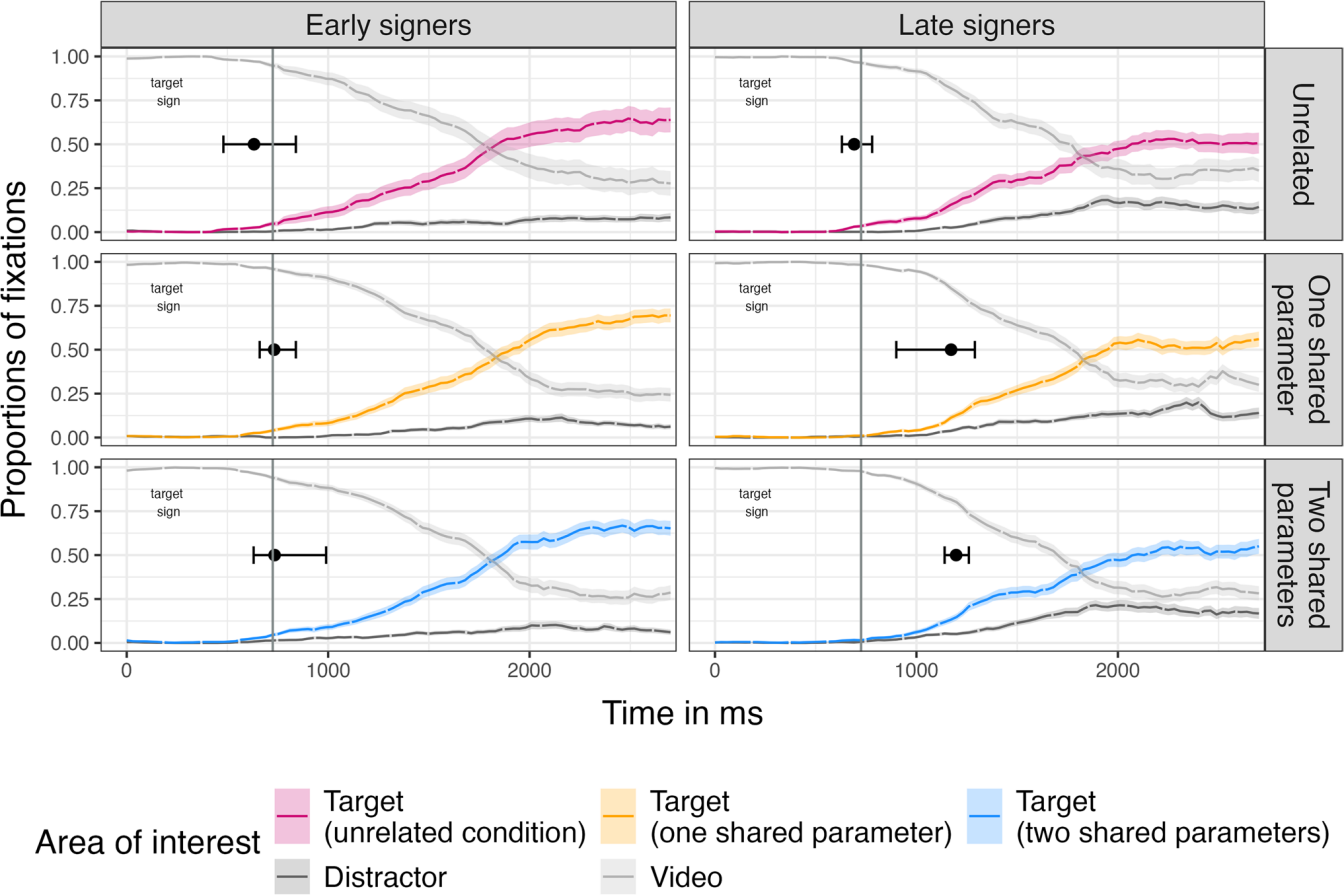
Time course of mean fixations for early signers (left column) and late signers (right column) to the video, target picture and distractor picture from target sign onset and 2000 ms following target sign offset. Conditions are presented as rows (from top to bottom): unrelated, one shared parameter and two shared parameters. The vertical solid line at 725 ms marks the target sign offset. The black dot marks the divergence point where fixations are significantly different between target and distractor, and whiskers indicate the confidence interval. Ribbons indicate standard error

We first plotted the time course of fixations to the video, target picture and distractor pictures by group for each condition in order to visually inspect looking patterns (Fig. 4).

##### Divergence point analysis

We predicted that early signers would show a significant divergence earlier than late signers, and that targets with phonologically related primes would be recognized earlier than targets with unrelated primes. As participants began each trial by watching the video, we considered the point at which the ratio of target to distractor looks exceeded zero to be the most sensitive measure for identify priming effects and allowed us to determine when the target was identified across groups and conditions. We first ran separate analyses using a bootstrapping approach comparing early and late signers in each condition (Table 6). Analysis revealed that the confidence interval surrounding the divergence point in the one shared parameter condition and the two shared parameter condition did not contain zero, indicating a reliable difference between groups with an earlier divergence point for early signers in both conditions. Groups did not differ significantly in the unrelated condition (For a detailed overview of the results, see the Supplemental Material B on OSF link (10.17605/OSF.IO/ESVR2).

**Table 6:** Group differences in target recognition by condition: Milliseconds refer to the divergence points for early and late signers in each condition and the mean difference in divergence points for early vs late signers and the 95 % confidence interval (CI). Positive mean difference indicate earlier divergence in early signers. Significant group differences are noted in bold

Condition	Early signers	Late signers	Mean difference between groups	95 % CI
Unrelated	638 ms	692 ms	54 ms	[-180, 240]
One shared parameter	732 ms	1174 ms	**442 ms**	**[151, 600]**
Two shared parameters	735 ms	1199 ms	**464 ms**	**[181, 600]**

Next, we ran separate analyses for each group using a bootstrapping approach (Ito & Knoeferle, 2023; Stone et al., 2021) to determine whether early or late signers had different fixation patterns based on the degree of phonological relatedness (Table 7). Early signers showed no difference in the timing of target between conditions, suggesting that they recognized the target along the same timeline regardless of priming condition. In contrast, late signers demonstrated later divergence points in the one shared and the two shared parameter condition compared to the unrelated condition. Thus, late signers were slower to recognize the target when preceded by a prime that shared any amount of phonological information.

**Table 7:** Within group comparisons for each condition: Mean difference in divergence points and the 95 % confidence interval (CI) for early and late signers for each pair of experimental conditions. Significant differences between conditions are noted in bold

Conditions	Early signers		Late signers	
	Mean difference between conditions	CI	Mean difference between conditions	CI
Unrelated vs one shared	98 ms	[-150, 300]	**484 ms**	**[240, 630]**
Unrelated vs two shared	105 ms	[-120, 360]	**505 ms**	**[390, 600]**
One shared vs two shared	2 ms	[-179, 240]	31 ms	[-150, 300]

##### Window analysis

**Fig. 5:**
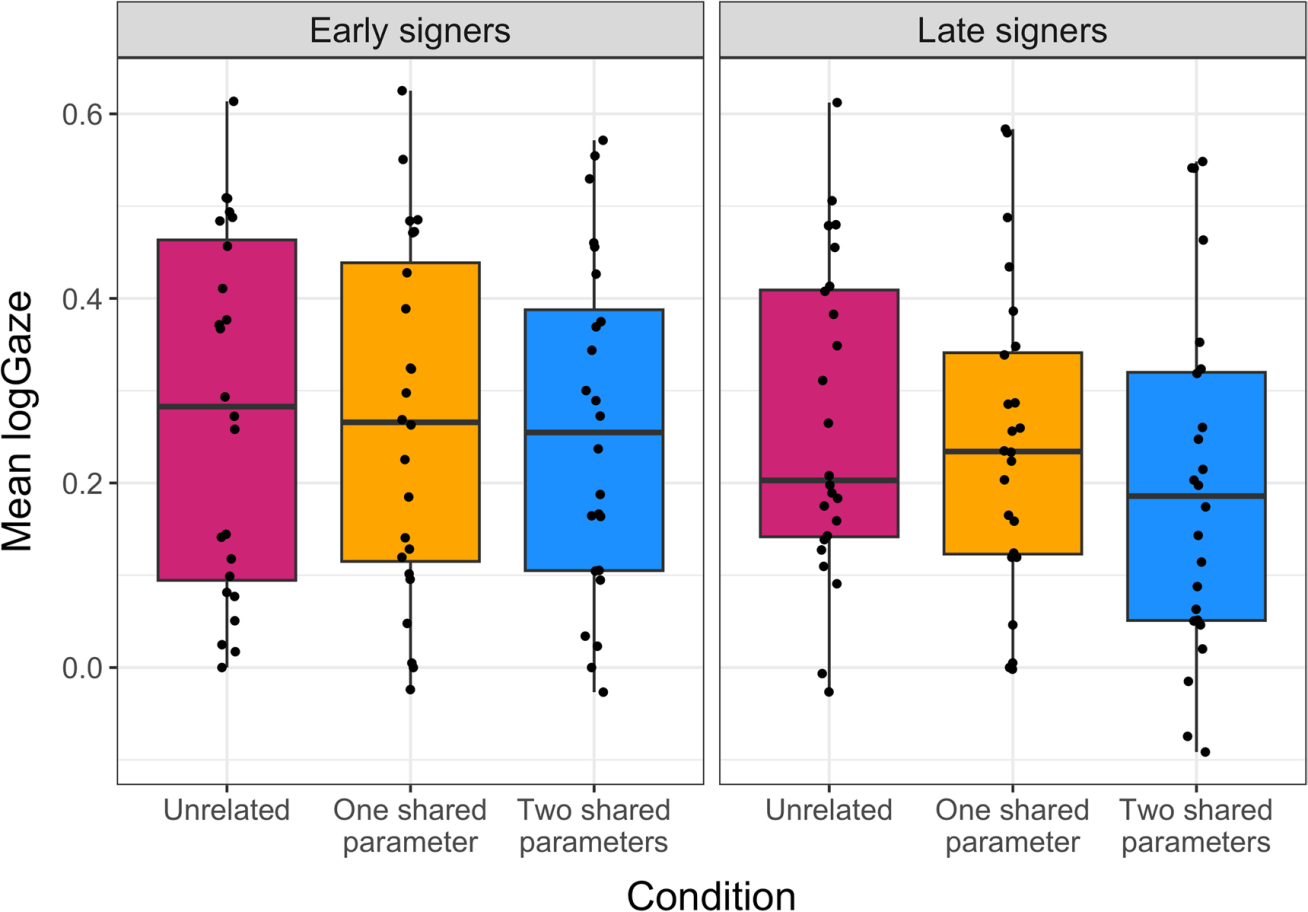
Mean logGaze of target vs distractor fixations for each group (left: early signers, right: late signers) and condition (on the x-axis from left to right: unrelated – pink, one shared parameter – orange, two shared parameters – blue). Dots indicate individual performance

In addition to the timing of divergence points, we also predicted that the proportion of fixations to the target would differ by group and condition across a longer recognition time window. We compared target to distractor looks by calculating mean log gaze ratios for the target picture to the distractor picture (Fig. 5), using the time window starting 200ms after target sign offset and continuing for 1000ms. We ran a mixed-effects model with log-transformed target vs. distractor looks as the outcome measure, and with group (early, late) and condition (unrelated, one shared, two shared) as well as their interaction as predictors, with participants and items as random effects. The factor condition was contrast-coded using the unrelated condition as the baseline. The analysis showed no significant effect of group and condition and no interaction (all p > .2). Thus, signers demonstrated similar overall fixations regardless of age of ASL acquisition and degree of phonological relatedness between the prime and target signs.

##### Interim summary

Both groups were highly accurate in selecting the target irrespective of degree of relatedness, but early signers were faster overall than late signers. The groups organized gaze differently and differed in how quickly they fixated the target with earlier fixations in early than in late signers when sign pairs shared phonological information. Early signers were not affected by the presence of any relatedness within the sign pairs and were not sensitive to the amount of relatedness. In contrast, late signers were affected by the relatedness between the prime and target but were not sensitive to the amount of relatedness.

#### Type of relatedness

We asked how signers varied in gaze patterns based not only on how many parameters are shared, but which specific parameters are shared. The goal of this analysis was to identify whether specific phonological parameters or combinations of parameters shared between prime and target signs are driving the overall difference in phonological processing, and whether this differed for early vs late signers. This analysis is exploratory, in that we did not predict a specific pattern of effects. For this analysis, we looked at early and late signers separately to determine whether specific related parameter contribute to facilitation or inhibition effects. We did not attempt to compare groups, because the difference in the time course of target recognition for phonologically related conditions between groups would hinder meaningful comparisons.

##### Divergence point analysis

We analyzed fixations to the target and distractor picture based on each of the six possible types of parameter relatedness (HS, MOV, LOC, HS+MOV, HS+LOC, MOV+LOC). We used a bootstrapping approach to determine significant divergences when fixations in each of the six phonological parameter conditions differed from fixations in the matched unrelated condition. We first tested for differences between groups for each phonological parameter condition. Analysis revealed that early signers were faster to recognize the target than late signers, but only when the prime sign shared location with the target sign (Table 8). A similar trend was observed for sign pairs sharing the same handshape.

**Table 8:** Comparison between groups: Divergence points for early and late signers in each parameter condition as well as the mean difference in divergence points for early vs late signers and the 95 % confidence interval (CI). Positive mean difference indicate earlier divergence in early signers

Parameter	Early signers	Late signers	Mean difference	95 % CI
Handshape	881 ms	1309 ms	**428 ms**	**[0, 690]**
Location	772 ms	1433 ms	**661 ms**	**[270, 869]**
Movement	1091 ms	1305 ms	214 ms	[-120, 720]
Handshape+Movement	1199 ms	1418 ms	219 ms	[-90, 810]
Handshape+Location	1160 ms	1634 ms	474 ms	[-210, 1140]
Location+Movement	971 ms	1197 ms	226 ms	[-210, 600]

**Fig. 6:**
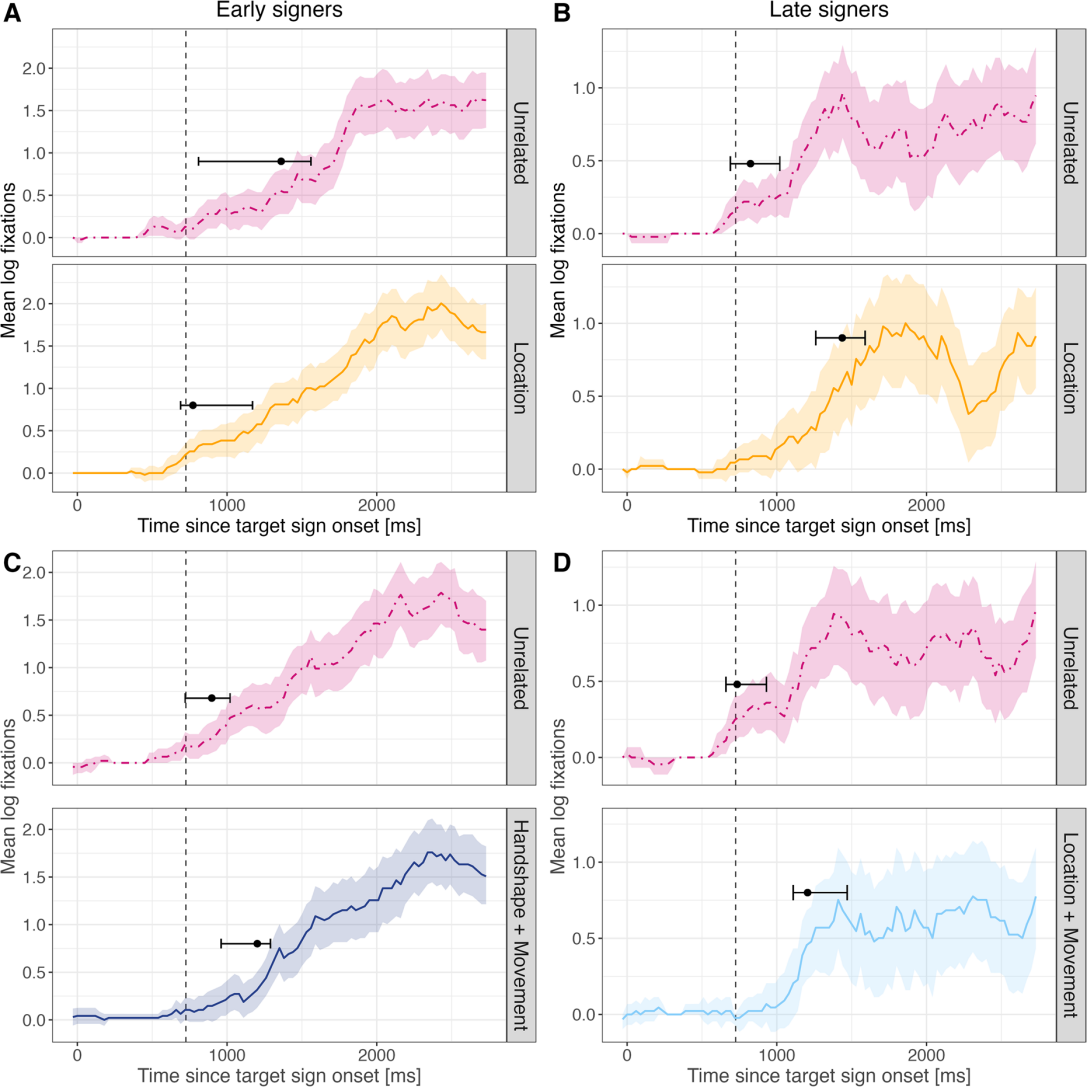
Mean log fixations where positive values indicate target fixations for early (**A** and **C**) and late signers (**B** and **D**) comparing the matched unrelated condition (pink, dotted line) and location (orange), handshape and movement (dark blue) and location and movement (light blue) from target sign onset and 2000 ms following target sign offset. The vertical dashed line at 725 ms marks the target sign offset. The black dot marks the divergence point where fixations in each parameter are significantly different from zero, and whiskers indicate the confidence interval. Ribbons indicate standard error

Next, we analyzed each group separately to determine whether early and late signers were sensitive to sign pairs that shared specific parameter(s) (Table 9). Among early signers, a pattern emerged in which signers were faster to recognize the target when it was primed by a phonologically related sign that shared location with the target, suggesting at least a partial facilitation effect of location. However, when sign pairs shared both handshape and movement, early signers demonstrated an inhibition effect. In contrast, late signers were consistently slower to recognize the target in the phonologically related conditions compared to the unrelated condition. This effect was significant for pairs that shared location, as well as location and movement, suggesting an inhibition effect. In Fig. 6, we plot the time course and divergence points for only those conditions where significant effects were observed.

**Table 9:** Within groups: Mean difference in divergence points and the 95 % confidence interval (CI) comparing each parameter condition to matched unrelated trials for early and late signers separately. Positive values indicate an earlier divergence point in the unrelated condition and negative values indicate an earlier divergence point in the parameter condition

	Early signers		Late signers	
Conditions	Mean difference between condition and Unrelated baseline	Confidence interval	Mean difference between condition and Unrelated baseline	Confidence interval
Handshape	-43 ms	[-300, 240]	97 ms	[-330, 599]
Location	**-**589 ms	**[-**810, -30**]**	613 ms	**[**360, 840**]**
Movement	192 ms	[-60, 510]	399 ms	[-240, 930]
Handshape+Movement	295 ms	**[**30, 510**]**	413 ms	[-180, 1022]
Handshape+Location	47 ms	[-540, 630]	655 ms	[0, 1230]
Location+Movement	-44 ms	[-420, 689]	471 ms	**[**240, 750**]**

##### Window analysis

**Fig. 7:**
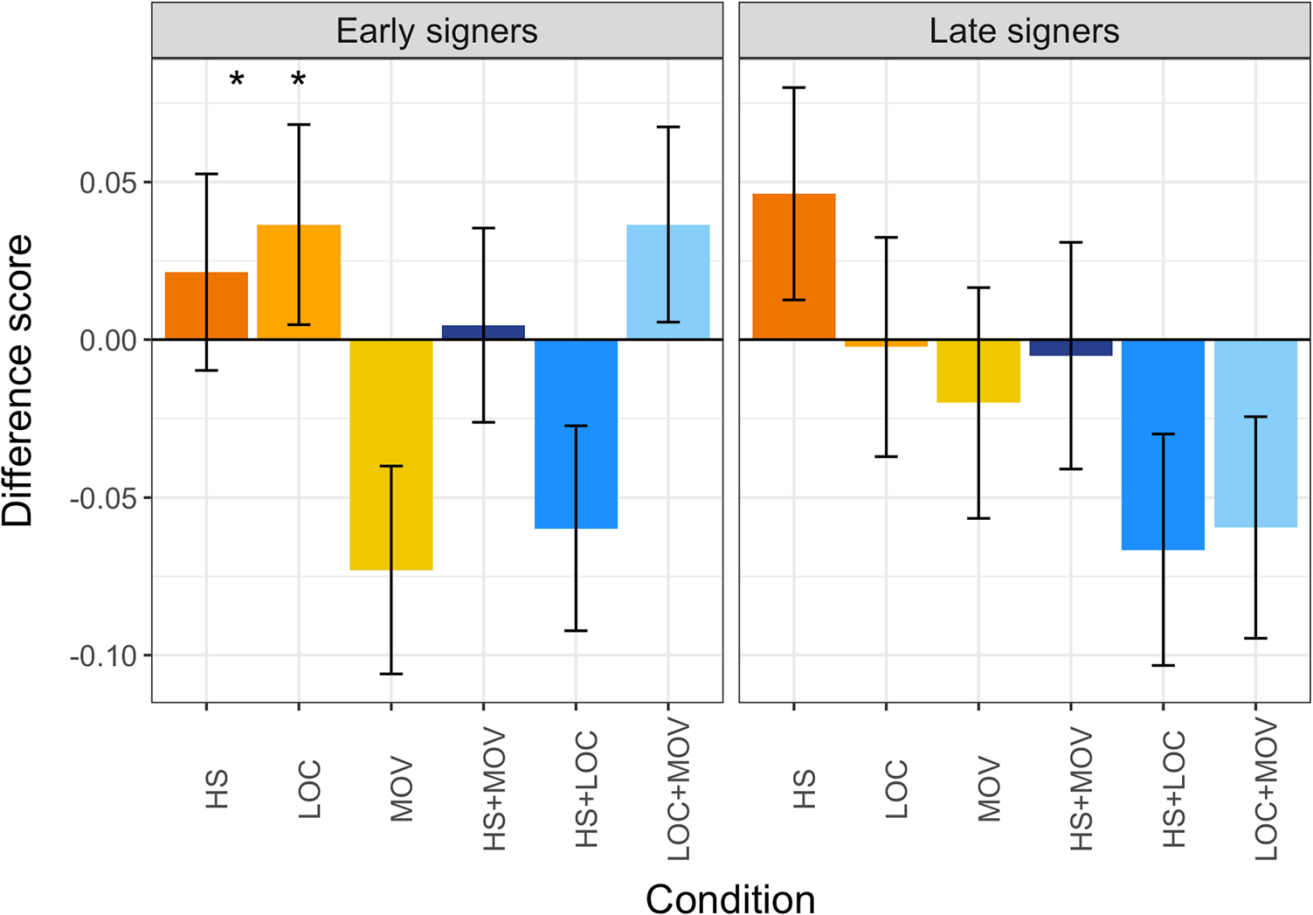
Mean difference score for early signers (left column) and late signers (right column) and each parameter (dark orange: handshape, orange: location, yellow: movement, dark blue: handshape and movement, blue: handshape and location, light blue: location and movement). Asterisks mark significance levels (* *p* < .05). Error bars indicate standard error

To assess facilitation or inhibition effects, we computed a difference score for the log gaze probabilities for each parameter by subtracting the log ratios in the matched unrelated condition (i.e., the baseline) from the log ratios in each condition (Fig. 7). Again, we used the time window starting 200ms after target sign offset and continuing for 1000ms. Facilitation is indicated by positive values and inhibition is reflected in negative values while a difference score of zero would indicate no effect.

Visual inspection suggested high variability in difference scores among the different parameters and parameter combinations. As Shapiro-Wilks tests showed non-normal distributions of the data (all p < .001), one-sample Wilcoxon signed rank tests were applied to test whether the difference score for each parameter statistically deviated from zero. Early signers showed inhibition for the shared movement parameter and for the shared combination of handshape and location. Late signers did not exhibit any facilitation or inhibition effects (Table 10).

**Table 10:** One-sample Wilcoxon signed-rank tests comparing the difference score against zero for each group and parameter. The table shows the z-value, the p-value and the effect size Cohen’s d. Asterisks mark the significant level

Parameter	Early signers			Late signers			
	*z*	*p*	*d*	*z*		*p*	*d*
HS	.67	.50	.08	1.35		.18	.12
LOC	.97	.33	.09	-.16		.87	.01
MOV	-2.01	.04*	.13	-.55		.58	.06
HS+MOV	.16	.88	.02	-.25		.80	.01
HS+LOC	-2.08	.04*	.13	-1.44		.15	.11
LOC+MOV	1.30	.19	.08	-1.47		.14	.11

* *p* < .05

##### Interim summary

Both early and late signers showed parameter-based differences, but for different parameters. In early signers, we observed a facilitation effect in faster looks to the target in the time course for sign pairs with shared location, and an inhibition effect for sign pairs that shared handshape and movement. In late signers, we observed an inhibition effect with later fixations to the target when preceded by primes that shared location or location and movement relative to unrelated primes. The large variability within and across conditions suggests that effects observed for degree of phonological similarity are not driven by a particular parameter or parameter combination.

## Discussion

We conducted a phonological priming eye tracking task with deaf signers who varied in their age of acquisition of ASL. We presented signers with ASL sentences containing sign pairs that were either unrelated or phonologically related in all possible combinations of the phonological parameters handshape, location and movement. We manipulated the degree (i.e., number of shared parameters) and type (i.e., which parameters) of phonological relatedness to investigate their effects on sign recognition. With respect to degree of relatedness, early signers demonstrated an earlier divergence point between target and distractor looks than late signers in conditions where signs shared at least one parameter, suggesting an overall advantage for early language exposure in phonological sign processing. Contrary to our expectations, early signers did not show earlier target recognition for phonologically related signs compared to unrelated signs. In contrast, late signers took longer to recognize the target in the presence of phonological relatedness, showing a general inhibition effect of a phonologically related prime. With regard to the type of relatedness, there was a high degree of variability. Signs that shared a location with the prime were the most salient, but affected early and late signers in contrasting ways. Our results confirmed the advantage of early sign exposure on efficient sign processing overall, but did not reveal clear patterns regarding the parameter or parameter combinations that drive phonological processing. Below, we discuss how the current findings contribute to our overall understanding of sign processing by signers with diverse linguistic backgrounds.

### Age of ASL acquisition affects phonological processing efficiency

Many studies have documented processing differences between early and late signers and these differences are often attributed to less automatic processing in late signers (MacSweeney et al., 2008; for an overview, see Mayberry, 2010). In the current study, we observed an overall difference in the reaction time to select the target (via key press). As predicted, early signers identified the target faster than late signers overall. However, we speculate that the reaction time data was less sensitive overall than the eye-tracking data, so we focus our discussion on the more fine-grained differences in eye-tracking patterns. Here, early and late signers did not differ in time to identify the target when there was no phonologically related prime, but early signers were faster than later signers in shifting gaze to the target picture when the prime shared one or two parameters with the target. Thus, the presence of phonological primes had an inhibitory effect on lexical recognition, but only for signers with a late age of exposure to ASL.

Signers with later ages of ASL language acquisition attend more to the phonological form of signs, which inhibits their processing of sign meaning; Mayberry and Fischer (1989) describe this as a “phonological bottleneck.” The late signers may have been particularly sensitive to the phonological form of the prime and target signs and, thus, were slower to identify the target when there was similarity between the prime and target signs. Competition between phonological representations of the prime and target signs might also account for the effect of increasing phonological similarity for late signers. Thierfelder et al. (2020) studied the effects of phonological activation of signs in deaf adult signers in a reading task using a parafoveal preview paradigm. They found higher preview costs for identical previews relative to previews overlapping in two parameters suggesting that, with decreasing phonological similarity, preview costs decrease as well. Thierfelder and colleagues propose that increased overlap in prime-target pairs evokes greater activation of lexical representations. In their study, signers with later age of sign language acquisition benefitted less from identical previews during reading, suggesting that age of sign language acquisition modulates reading efficiency. Late signers may also require more information about a sign before they have sufficient information to recognize it and match it to a target picture. In gating tasks, late signers consistently identify a sign at a later gate than early signers (Emmorey & Corina, 1990; Morford & Carlson, 2011).

Early and late signers in our study likely differed in how they perceived phonological information. Signers with an earlier age of sign language acquisition demonstrate perceptual abilities that are more fine-tuned to linguistically relevant information than signers who did not have full exposure to language during the critical period. Our findings contrast with earlier findings in that early signers showed no differences in perception based on the presence or absence of a phonologically related prime sign. The lack of effect could partially be related to the approach of using phonological priming in a sentence context. Early signers’ processing is known to be highly efficient, thus the current paradigm might not be sensitive enough to detect more subtle differences. Alternatively, effects could be driven by specific parameter combinations, so that an analysis averaging across the different parameters could lead to blurring of effects. In previous work, age of sign language acquisition effects were not observed similarly for each phonological parameter; effects were mostly observed for handshape and were not observed for location (e.g., Morford et al., 2008) while movement was rarely investigated.

Meade et al. (2021) argue against phonological neighborhood density (i.e., number of competing neighboring signs) as an explanation for effects of degree of relatedness. As they presented the same target items in related and unrelated conditions, Meade et al. (2021) propose that the nature of connections between signs (i.e., type of relatedness, rather than the number of connections) is the most likely explanation for the observed effects. In the current study, phonological neighborhood density is not a plausible explanation for the effects of phonological similarity, because the targets were consistent (and thus had consistent phonological neighborhood density) across conditions, nor did primes differ in their phonological neighborhood density between conditions. The group differences can likely be attributed to the speed and efficiency with which early signers process and identify the target sign, and not to their ability to connect signs and their referents accurately.

### Phonological processing effects are driven by multiple parameters

We manipulated which parameter(s) the sign pairs shared and observed a high degree of variability across conditions in both early and late signers. Early signers showed faster looks to the target for sign pairs with shared location, and slower looks to the target for shared handshape and movement compared to matched unrelated sign pairs. In contrast, late signers showed slower looks to the target for sign pairs sharing location as well as location and movement compared to matched unrelated sign pairs. This points towards a special role of the location parameter which may be influencing signers in contrasting ways depending on their age of sign language acquisition. Previous studies of phonological processing suggest a consistent effect of location but are mixed with respect to its effect, with some showing a facilitation effect (Dye & Shih, 2006; Meade et al., 2021), and others showing inhibition (Carreiras et al., 2008; Corina & Emmorey, 1993; Corina & Hildebrandt, 2002; Gutiérrez et al., 2012; Meade et al., 2021) among early signers, and at least one prior study showing inhibition based on location among late signers (Gutiérrez et al., 2012). We speculate that the visual salience of location in the current task, in which the prime and target were separated by a filler sign, may have drawn signers to attend to location when processing the target sign. Potential characteristics of location affecting sign recognition could be perceptual saliency, which is shaped by language experience (Poizner, 1983) or its central role in identifying signs as the earliest recognizable parameter (Emmorey & Corina, 1990).

While the divergence point allows us to pinpoint the role of phonological relatedness in initial sign recognition, the window analysis lends itself to understanding overall effects in the robustness of sign recognition. Here, we observed fewer effects of specific parameters in late signers compared to early signers. This group difference cannot be attributed to overall perception difficulties in late signers. Morford & Carlson (2011) showed similar or even slightly better performance among late signers compared to early signers in a handshape and location monitoring task. Instead, early and late signers might exhibit different sensitivity to phonological information. Linguistic experience has been shown to affect phonological similarity judgements (Hall et al., 2012; Hildebrandt & Corina, 2002). This sensitivity seems not to apply only to the degree of phonological relatedness but also to the type of relatedness.

Although our current approach was designed to investigate phonological priming effects, there are additional strategies that signers may have used to identify the target sign. As the sentence unfolds, signers may have used more top-down processing approaches. For example, signers may have used the preview period to identify both pictures, and might have activated the signs corresponding to the pictures. Thus, signers might have narrowed down the cohort of potential target signs to the two presented pictures and then applied that knowledge to identify the target sign as the sentence unfolded. This pre-activation of signs might affect early and late signers differently. Indeed, in prior work examining signers perceiving ASL sentences, we found evidence that semantic information in the sentence supports early lexical recognition (Lieberman et al., 2018; Wienholz & Lieberman, 2019). It is unlikely that this type of approach could fully explain the current results, because the sentences did not provide any semantically constraining information. However, we cannot rule out the possibility that signers are bringing a range of approaches to the task, including both bottom-up and top-down processing strategies

## Limitations

This study comes with some limitations that might have concealed potential effects. With respect to the behavioral task, using a cue (i.e., a question mark), that occurred after an extended time with a delay, might have led to ceiling effects in accuracy and reaction time. Therefore, the reaction time data are less sensitive to reveal potential effects. Furthermore, we presented full ASL sentences, which is closer to natural language use than presenting single signs. The length of time between the prime and target sign, as well as the need to direct gaze to the video before shifting gaze to the pictures, may have reduced the effects of phonologically related primes. Further studies using a range of approaches are needed to better understand whether phonological relatedness impact processing among signers with various backgrounds. Specifically, a design that more tightly couples prime and target, or allows for simultaneous viewing of signs and pictures, might provide a clearer measure of phonological activation.

## Conclusion

Our findings support previous claims that sub-lexical features impact phonological processing in ASL signers, and that signers who acquired ASL after early childhood show persistent effects of delayed language exposure. Both early and late signers were sensitive to phonological information when processing ASL sentences in their timing, but not overall fixations of target signs. Late signers were slower overall to recognize the target sign, particularly when preceded by a prime that was phonologically related to the target sign. We show that phonological effects persist when processing signs embedded in sentences. Our findings also highlight the need to carefully control the degree and type of phonological relatedness when studying sign processing. This study, while leaving open questions about the nuances of phonological processing in sign language, adds to the growing body of evidence that early language exposure is critical for efficient language processing.

## Data Availability

The datasets generated during and/or analyzed during the current study are available in the OSF repository, 10.17605/OSF.IO/ESVR2.

## Footnotes

1 Notational conventions: signs are glossed in small caps. index denotes a pointing sign, typically produced using the extended index finger.

2 Half of the distractors were targets from other sign pairs while the other half were non-target distractors; in this way participants could not guess the target solely based on the presented pictures from previous trials.

3 Filler signs had a mean length of 863 ms with a range of 640 ms to 1200 ms. T-tests showed no difference when comparing mean length of time between conditions (all p > .14).

## Code availability

The code used for data analyses and data visualizations are available in the OSF repository, 10.17605/OSF.IO/ESVR2.
